# C-reactive protein and procalcitonin to discriminate between tuberculosis, *Pneumocystis jirovecii* pneumonia, and bacterial pneumonia in HIV-infected inpatients meeting WHO criteria for seriously ill: a prospective cohort study

**DOI:** 10.1186/s12879-018-3303-6

**Published:** 2018-08-14

**Authors:** Fiona Mendelson, Rulan Griesel, Nicki Tiffin, Molebogeng Rangaka, Andrew Boulle, Marc Mendelson, Gary Maartens

**Affiliations:** 1 School of Public Health and Family Medicine, University of Cape Town, Cape Town, South Africa; 2 Division of Clinical Pharmacology, Department of Medicine, University of Cape Town, Cape Town, South Africa; 3 Centre for Infectious Diseases Research Initiative, Institute of Infectious Diseases & Molecular Medicine, University of Cape Town, Cape Town, South Africa; 4 Department of Infection & Population Health, Institute of Global Health, University College London, London, UK; 5 Department of Medicine, University of Cape Town, Cape Town, South Africa; 6 Division of Infectious Diseases and HIV Medicine, Department of Medicine, University of Cape Town, Cape Town, South Africa

**Keywords:** HIV, Tuberculosis, Bacterial community-acquired pneumonia, *Pneumocystis jirovecii* pneumonia, C-reactive protein, Procalcitonin, Diagnostic accuracy, WHO algorithm

## Abstract

**Background:**

Tuberculosis, bacterial community-acquired pneumonia (CAP), and *Pneumocystis jirovecii* pneumonia (PJP) are the three commonest causes of hospitalisation in HIV-infected adults. Prompt diagnosis and treatment initiation are important to reduce morbidity and mortality, but are hampered by limited diagnostic resources in resource poor settings. C-reactive protein (CRP) and procalcitonin have shown diagnostic utility for respiratory tract infections, however few studies have focussed on their ability to distinguish between tuberculosis, CAP, and PJP in HIV-infected inpatients.

**Methods:**

We evaluated the diagnostic accuracy of CRP and procalcitonin, compared with composite reference standards, to discriminate between the three target infections in adult HIV-infected inpatients in two district level hospitals in Cape Town, South Africa. Participants were admitted with current cough and danger signs in accordance with the WHO algorithm for tuberculosis in seriously ill HIV-infected patients. Study clinicians were blinded to CRP and procalcitonin results.

**Results:**

Two hundred forty-eight participants met study case definitions: 133 with tuberculosis, 61 with CAP, 16 with PJP, and 38 with mixed infection. In the 210 particpants with single infections the differences in median CRP and procalcitonin concentrations between the three infections were statistically significant, but distributions overlapped considerably. CRP and procalcitonin concentrations were highest in the CAP group and lowest in the PJP group. CRP and procalcitonin cut-offs with sensitivities of ≥90% were found for all three target infection pairs, but corresponding specificities were low. Highest receiver operating characteristic areas under the curve for CRP and procalcitonin were for PJP versus tuberculosis and PJP versus CAP (0.68 and 0.71, and 0.74 and 0.69 respectively).

**Conclusions:**

CRP and procalcitonin showed limited value in discriminating between the three target infections due to widely overlapping distributions, but diagnostic accuracy was higher for discriminating PJP from CAP or tuberculosis. Our findings show limitations for CRP and procalcitonin, particularly for discriminiation of tuberculosis form CAP, however they may have greater diagnostic utility as part of a panel of biomarkers or in clinical prediction rules.

**Electronic supplementary material:**

The online version of this article (10.1186/s12879-018-3303-6) contains supplementary material, which is available to authorized users.

## Background

Respiratory infections are a major cause for hospital admission in HIV-infected people globally in the antiretroviral era; the commonest being tuberculosis, bacterial community-acquired pneumonia (CAP), and *Pneumocystis jirovecii* pneumonia (PJP) [1]. Prompt diagnosis and initiation of appropriate treatment is important to reduce mortality in HIV-infected inpatients.

Determining the aetiology of serious infections in inpatients with HIV is challenging, in part due to considerable overlap in the clinical and radiographic presentation of tuberculosis, CAP, and PJP [2]. Atypical presentation and dual infection further compound these diagnostic challenges. Furthermore, there are limitations of current diagnostic methods and limited access to diagnostic tests in resource poor settings [3–5].

WHO’s algorithm for the diagnosis of tuberculosis in seriously ill patients [6] recommends broad spectrum antibiotics, that treatment for PJP should be considered (without giving guidance on selection of patients for empiric PJP therapy), using a rapid nucleic acid amplification test (the Xpert MTB/RIF assay) to diagnose tuberculosis, and empiric therapy for tuberculosis if the Xpert MTB/RIF assay is negative or unavailable, and there is no response to antibiotics. Our group recently reported that 91.5% of patients defined as seriously ill by WHO and who had a current cough were diagnosed with tuberculosis, CAP, and/or PJP [7]. Incorporating simple affordable tests in clinical algorithms to discriminate between these three infections could improve outcomes in seriously ill patients. Two inflammatory biomarkers, C-reactive protein (CRP) and procalcitonin, have shown some diagnostic utility for bacterial respiratory infections [8, 9], and CRP has high sensitivity but low specificity for diagnosing HIV-associated tuberculosis [8]. An additional advantage is that both CRP and procalcitonin are available as affordable point-of-care tests [10, 11]. However, only two studies have examined diagnostic performance of CRP in discriminating between tuberculosis, CAP, and PJP in hospitalised patients with HIV, with conflicting results [2, 13]. We were unable to find any studies reporting diagnostic accuracy of procalcitonin in discriminating between all three infections.

The purpose of this study was to explore the diagnostic accuracy of CRP and procalcitonin in predicting presence or absence of each of the three major infections in seriously ill, HIV-infected inpatients. Secondary objectives were to describe the extent to which CRP and procalcitonin concentrations differed between the three infections and to determine optimal concentration cut-offs for discriminating those with and without each target infection.

## Methods

### Study setting and participants

We conducted a secondary analysis of a data from a large prospective cohort study [7], which was designed to improve the evidence base for the WHO algorithm for the diagnosis of tuberculosis in seriously ill HIV-infected participants with current cough [3]. Recruitment for the main study took place at two secondary level hospitals in Cape Town, South Africa, serving communities with high HIV and tuberculosis prevalence: G.F. Jooste District Hospital from November 2011 until the hospital’s closure in February 2013, and Khayelitsha District Hospital from March 2013 until October 2014.

Inclusion criteria for the main study were: admission into the enrollment facility within the previous 24 h, ≥18 years of age, known HIV infection, cough of any duration, and at least one WHO-defined danger sign (respiratory rate > 30/min, fever > 39^o^ C, pulse rate > 120/min, and unable to walk unaided). Exclusion criteria were: anti-tuberculosis treatment that was current, completed in the previous month, or defaulted in the past 6 months (isoniazid preventive therapy was allowed); exacerbation of either congestive cardiac failure or chronic obstructive pulmonary disease; and failure to provide a spontaneous or induced sputum specimen.

For the current study we added the inclusion criterion of a CRP and procalcitonin result, (funding for these two assays only became available after the start of the main study). We also only included participants fulfilling our a priori case definitions for tuberculosis, CAP, and/or PJP.

### Case definitions

Tuberculosis: positive *Mycobacterium tuberculosis* culture from any site plus at least one symptom consistent with tuberculosis (cough, fever, night sweats, weight loss). CAP: cough ≤14 days plus one or more additional respiratory symptoms (sputum, breathlessness, chest pain, haemoptysis or fever) plus radiological evidence of pulmonary consolidation (confirmed by a radiologist) [14]. PJP: cough ≤ 3 months plus radiological evidence of diffuse bilateral interstitial infiltrates (confirmed by a radiologist) plus oxygen saturation ≤ 92% (adapted from Centers for Disease Control and Prevention case definition) [15].

### Investigations

Three sputum specimens were obtained from each participant. One sample was sent for Gram stain, culture, and sensitivity, and two samples for smear examination with auramine staining for acid-fast bacilli (AFB) and liquid mycobacterial culture (BACTEC™ MGIT™ 960; Becton, Dickinson and Company, New Jersey, USA). Mycobaterial blood culture was done on all participants. Mycobacterial culture was done on other specimens when appropriate (e.g. pleural fluid).

CRP (Siemens Advia 1800), procalcitonin (Siemens Advia Centaur XP), and β-D-glucan assay (Fungitell™; Associates of Cape Cod, Inc., east Falmouth, MA, USA) were done on stored serum in a batch after the study, therefore these tests had no role in patient management. Laboratory staff were blinded to participant diagnosis and outcome. Assay range for CRP was 4-[304–336] mg/L, normal range was below 10 mg/L. Assay range for procalcitonin was < 0.02–75 μg/L, normal range below 0.02 μg/L.

Chest radiographs were performed on admission and retrospectively reviewed by a senior radiologist blinded to diagnoses and results of laboratory investigations.

### Statistical analyses

All analyses were performed using Stata software version 13.0 (StataCorp Inc., College Station, Texas, USA).

Based on our fixed sample size of 210 participants with single respiratory infections, we explored precision to detect 90% sensitivity for each biomarker for the three target infections, aiming for a maximum ±10% variation in 95% confidence intervals (CIs). We estimated a range of CIs of binomial proportions using the Wilson-score interval for smaller sample sizes [16]. Since our data was not normally distributed, a second calculation was made using 85% of the original sample sizes as suggested by Lehmann et al. [17]. We estimated the 95% CIs of 90% sensitivity to be 83–94% for tuberculosis, 79–96% for CAP, and 69–99% for PJP. The small sample size for PJP accounted for wide 95% confidence intervals. Further details of these sample size calculations are provided in an additional table (see Additional file 1: Table S1).

To detect differences in CRP concentrations between the three target infections, we estimated power for a two-sample means test (assuming unequal variances), based on relevant literature. (Expected means for CRP were approximate due to lack of reported standard deviations for tuberculosis or CAP). Our study had 80% power and alpha of 0.05 (using 85% of the original sample size to account for non-normal distribution of CRP and procalcitonin concentrations) to detect a minimum mean concentration difference in CRP between tuberculosis and PJP of 36%, between CAP and PJP of 14%, and between CAP and tuberculosis of 14%, and a minimum mean concentration difference in procalcitonin of 50% between tuberculosis and PJP, 62% between CAP and PJP, and 62% between CAP and tuberculosis. We were unable to find data on sensitivity estimates for procalcitonin in all three target infections in HIV-infected individuals, therefore calculations were based exclusively on studies reporting CRP measures of diagnostic accuracy. Further details of these sample size calculations are provided in an additional table (see Additional file 1:, Table S2).

Diagnostic accuracy analyses for CRP and procalcitonin were performed for participants fulfilling criteria for one of the three single infection definitions. Participants with mixed infection were analysed separately. In clinical practice, differential diagnostic challenges usually present between two of the target infections and less commonly between all three, hence we calculated diagnostic accuracy measures between infection pairs in addition to each target infection versus the other two.

As distributions of both CRP and procalcitonin were not normally distributed, we used non-parametric statistical tests for continuous variables. Univariate associations between participant baseline characteristics in infection pairs were analysed using the Wilcoxon-Mann-Whitney test for continuous data, and Chi-square test (or Fisher’s Exact test if values in a cell were < 5), for categorical data. All probability tests were two-tailed. CRP and procalcitonin values below the detectable limit (BDL) of the assay were substituted with half BDL (in preference to substitution with the assay limit or with zero, both of which have been shown to bias parameter estimates) [18].

Receiver Operating Characteristic (ROC) area under the curve (AUC) analyses were used to explore potential cut-offs for CRP for each target infection using Liu’s index [19], which we then used to calculate diagnostic accuracy estimates. Cut-offs were also explored using the WHO 90% sensitivity recommendation for screening tests for tuberculosis [20]. To mitigate overfitting and improve accuracy of model prediction, we performed cross-validation on all ROC AUC’s exceeding 60% using k-fold cross-validation, as the dataset was too small for generation of a training set.

Since there are few studies on the diagnostic accuracy of procalcitonin for infections in HIV-infected patients, we explored cut-offs established for both lower respiratory tract infections (LRTI) and sepsis. Procalcitonin categories for LRTI were: < 0.1 μg/L, bacterial infection very unlikely; 0.1–0.25 μg/L, localised bacterial infection unlikely; 0.25–0.5 μg/L, localised bacterial infection possible; > 0.5 μg/L, suggestive of bacterial infection. Procalcitonin categories for systemic bacterial infection / sepsis were: 0.5–2 μg/L, systemic infection possible; 2–10 μg/L, suggestive of systemic infection; > 10 μg/L, severe systemic infection / septic shock [9].

This study conforms to the Standards for Reporting Diagnostic Accuracy Studies (STARD) guidelines [21].

## Results

### Participant characteristics

**Fig. 1:**
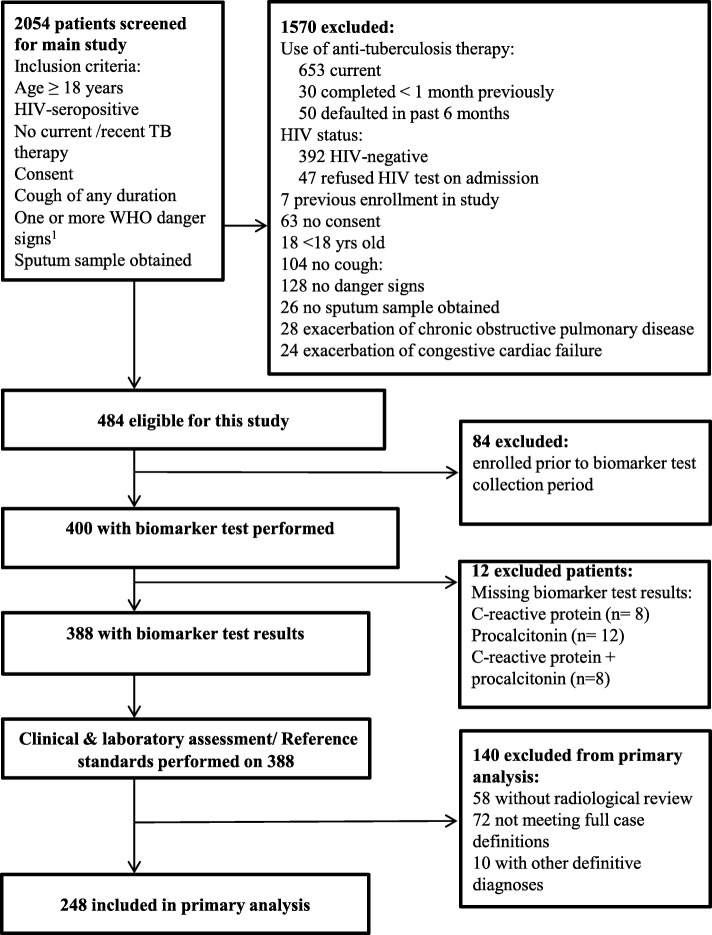
Consort diagram based on Standards for Reporting Diagnostic Accuracy Studies (STARD)

**Fig. 2:**
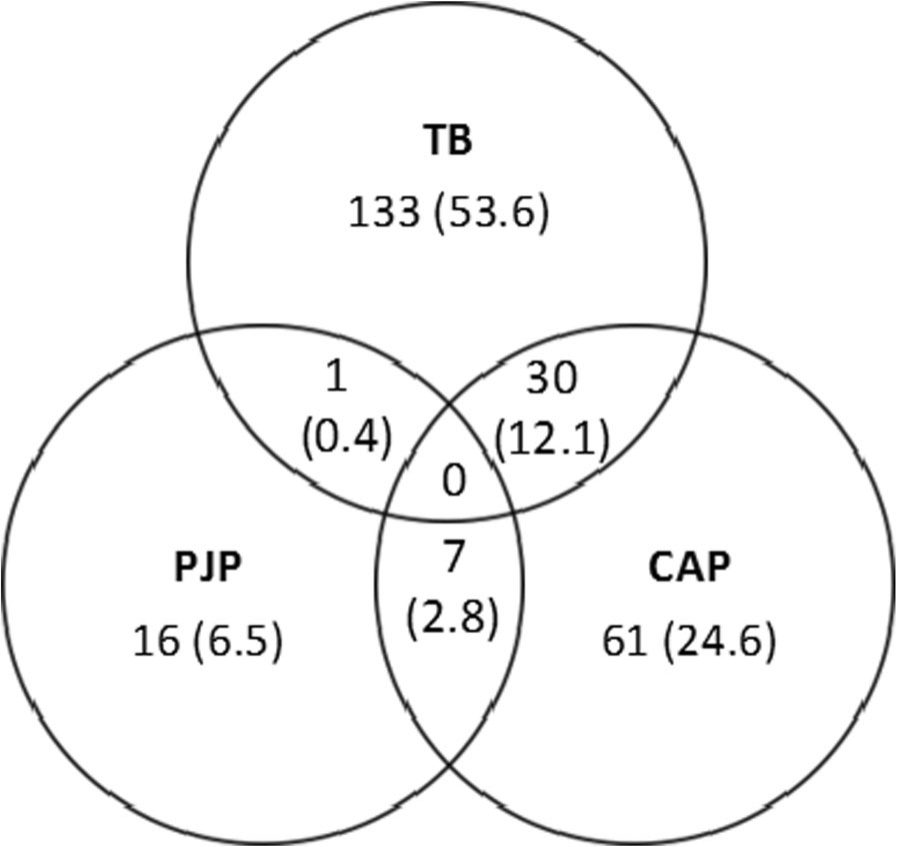
Venn diagram of number of participants diagnosed with single target infections and mixed infections. Numbers in parentheses are %

The participant flow diagram is shown in Fig. 1. Ten participants were excluded due to diagnoses other than the three target infections (meningitis, disseminated *Cryptococcus,* emmonsia, bronchitis, and post-TB bronchiectasis with a pneumothorax). All participants had received at least one dose of antibiotics as per WHO guidelines (usually ceftriaxone) prior to obtaining blood samples used for biomarker tests. 73/248 (29%) reported taking cotrimoxazole prophylaxis prior to admission. Figure 2 summarises the distribution of target infections in all 248 participants.

Baseline characteristics of the 210 paticipants with one of the three single infection diagnoses (i.e. excluding participants with mixed infection) are shown in Table 1. Tuberculosis was associated with lower haemoglobin concentrations and reported weight loss compared with the other two groups. Inability to walk unaided was more common in those with tuberculosis than in those with CAP. Those with CAP had higher median white cell count and CD4 count than the other two infections. The CAP group was more likely to have been using antiretroviral therapy prior to admission compared with the tuberculosis group. Participants with PJP were much more likely to have β-D-glucan concentrations exceeding 300 pg/L, had lower CD4 counts, and were more likely to have a respiratory rate above 30/min than participants in the other two groups.

**Table 1:** Baseline characteristics of 210 participants with a single target infection by infection status

Diagnosis n (%)	Total *N* = 210	TB *n* = 133 (63)	CAP *n* = 61 (29)	PJP *n* = 16 (8)	*P*-value for pairwise comparison^a^
Median age in yrs. (IQR)	34.8 (28.9–40.7)	34.7 (29.1–40.8)	35.1 (29.4–40.0)	36.9 (28.8–41.2)	TB vs. CAP: 0.96, CAP vs. PJP: 0.84
					PJP vs. TB: 0.75
Sex: female n(%)	139 (66)	84 (63)	44 (72)	11 (69)	TB vs. CAP: 0.22, CAP vs. PJP: 0.71^b^
					PJP vs. TB: 0.79^b^
Cotrimoxazole prophylaxis	60 (29)	38 (29)	18 (30)	4 (25)	TB vs. CAP: 0.89, CAP vs. PJP: 0.49^b^
					PJP vs. TB: 1.00^b^
Antiretroviral therapy n (%)	76 (36)	43 (32)	29 (48)	4 (25)	TB vs. CAP: 0.04, CAP vs. PJP: 0.16^b^
					PJP vs. TB: 0.78^b^
Median CD4^+^ count, cells/μL (IQR)	97 (38–210)	77 (35–162)	200 (79–287)	35 (12–81)	TB vs. CAP: 0.0001
					CAP vs. PJP: 0.0005, PJP vs. TB: 0.03
Median WCC ×10^9^/L (IQR)	8.6 (5.8–12.9)	7.3 (5.2–10.2)	12.3 (8.4–20.0)	8.2 (6.2–10.7)	TB vs. CAP: 0.0001, CAP vs. PJP: 0.01
					PJP vs. TB: 0.5
Median Hb g/dL (IQR)	9.4 (7.7–10.8)	8.6 (7.4–10.1)	10.4 (8.8–12)	11.25 (9.7–12.2)	TB vs. CAP: 0.0001, CAP vs. PJP: 0.24
					PJP vs. TB: 0.0001
β-D-glucan > 300 pg/mL	25 (12)	11 (8)	1 (2)	13 (80)	TB vs. CAP: 0.11^b^, CAP vs. PJP: < 0.0001^b^ PJP vs. TB: < 0.0001^b^
WHO danger signs^c^:					
Pulse rate > 120 beats/min	166 (79)	106 (80)	51 (84)	9 (56)	TB vs. CAP: 0.52, CAP vs. PJP: 0.02
					PJP vs. TB: 0.04
Respiratory rate > 30/min	137 (65)	83 (62)	38 (62)	16 (100)	TB vs. CAP: 0.99, CAP vs. PJP: 0.002^b^
					PJP vs. TB: 0.001^b^
Fever> 39 °C	31 (15)	20 (15)	10 (16)	1 (6)	TB vs. CAP: 0.81, CAP vs. PJP: 0.44^b^
					PJP vs. TB: 0.47^b^
Unable to walk unaided	119 (57)	88 (67)	23 (38)	8 (50)	TB vs. CAP:< 0.0001, CAP vs. PJP: 0.40
					PJP vs. TB: 0.19
TB symptoms^d^:					
Night sweats	137 (66)	89 (67)	38 (63)	10 (67)	TB vs. CAP: 0.58, CAP vs. PJP:1.00^b^
					PJP vs. TB: 0.78^b^
Weight loss	196 (94)	130 (98)	53 (88)	13 (81)	TB vs. CAP: 0.005^b^, CAP vs. PJP: 0.43^b^
					PJP vs. TB: 0.009^b^
Fever	170 (82)	106 (80)	52 (87)	12 (75)	TB vs. CAP: 0.29, CAP vs. PJP: 0.27^b^
					PJP vs. TB: 0.74^b^

Abbreviations: *TB* tuberculosis, *CAP* bacterial community-acquired pneumonia, *PJP* Pneumocystis jirovecii pneumonia, *Hb* haemoglobin, *WCC* white cell count

^a^Hypothesis tests- Wilcoxon-Mann-Whitney test for continuous data; Chi-square test for categorical data. ^b^Fisher’s exact test where 1 or more cells < 5

^c^Danger signs based on WHO algorithm for diagnosis of TB in seriously ill patients; ^d^ Cough of any duration was a study inclusion criterion

### CRP concentrations and diagnostic utility for each infection

**Fig. 3:**
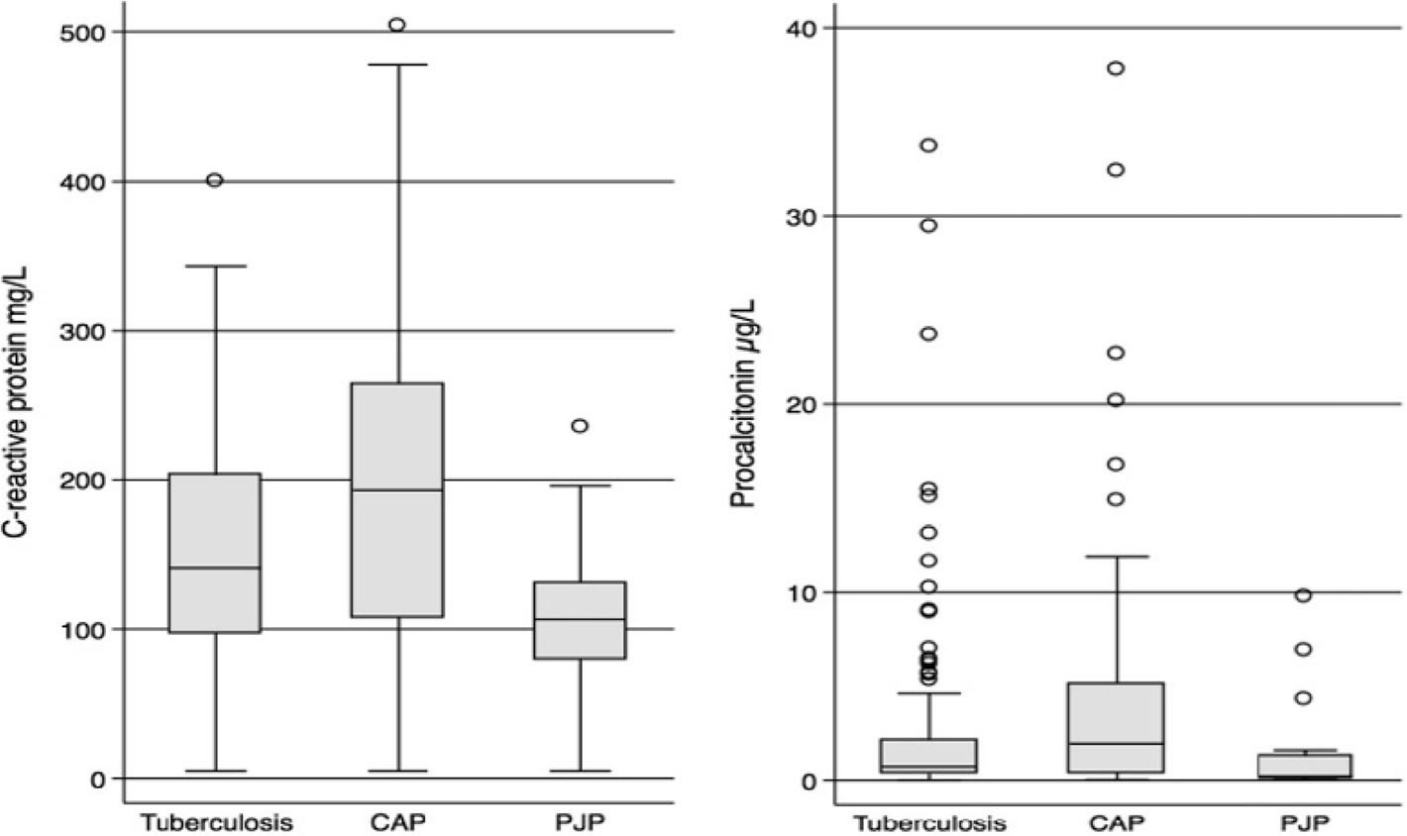
Distribution of (**a**) C-reactive protein and (**b**) procalcitonin in the 210 participants with single infections

Distributions of CRP concentrations by single infection category are shown in Fig. 3a. Comparison of CRP concentrations between the three single infections are shown in Table 2. Elevated CRP concentrations (> 10 mg/L) were found in 206/210 (98%) participants: 131/133 (98%) with tuberculosis, 60/61 (99%) with CAP, and 15/16 (94%) with PJP. There were statistically significant differences in median concentrations between infection pairs, with considerable overlap in distributions. The highest concentrations were in participants with CAP and the lowest concentrations in those with PJP.

**Table 2:** C-reactive protein and procalcitonin distributions by infection in 210 participants with a single target infection

Biomarker concentration	Total (*N* = 210)	TB (*n* = 133)	CAP (*n* = 61)	PJP (*n* = 16)	*P*-value ^c^
Median CRP mg/L (IQR)	148 (96–224)	141 (97–203)	193 (108–264)	106.5 (79.5–131.5)	TB vs.CAP: 0.02, CAP vs. PJP: 0.003
					PJP vs. TB: 0.02
CRP ≥ 10 mg/L^a^ n (%)	206 (98)	131 (98.5)	60 (98.4)	15 (93.8)	TB vs.CAP: 1.00^d^, CAP vs. PJP: 0.38^d^
					PJP vs. TB: 0.29^d^
Median PCT μg/L (IQR)	0.8 (0.3–2.9)	0.7 (0.4–2.1)	2.0 (0.4–5.2)	0.2 (0.1–1.3)	TB vs. CAP: 0.05, CAP vs. PJP: 0.01
					PJP vs. TB: 0.05
PCT ≥ 0.02 μg/L^b^: n (%)	209 (99.5)	132 (99)	61 (100)	16 (100)	–
PCT ≥ 0.1 μg/L: n (%)	199 (94.8)	128 (96.2)	58 (95.1)	13 (81.3)	TB vs. CAP: 0.71^d,^, CAP vs. PJP: 0.10^d^
					PJP vs. TB: 0.04^d^
PCT ≥ 0.25 μg/L: n (%)	170 (81)	112 (84)	50 (82)	8 (50)	TB vs. CAP: 0.70, CAP vs. PJP: 0.008
					PJP vs: TB: 0.001
PCT ≥ 0.5 μg/L: n (%)	137 (65.2)	87 (65.4)	43 (70.5)	7 (43.8)	TB vs. CAP: 0.49, CAP vs. PJP: 0.05
					PJP vs. TB: 0.11
PCT ≥ 2 μg/L: n (%)	69 (32.9)	36 (27.1)	30 (49.2)	3 (18.8)	TB vs. CAP: 0.003, CAP vs. PJP: 0.05^d^
					PJP vs. TB: 0.56^d^
PCT > 10 μg/L: n (%)	16 (8)	8 (6)	8 (13)	0 (0)	–

Abbreviations: *CRP* C-reactive protein, *PCT* Procalcitonin, *TB* tuberculosis, *CAP* bacterial community-acquired pneumonia, *PJP* Pneumocystis jirovecii pneumonia

^a^Elevated concentration. ^b^Lower detectable limit for procalcitonin assay

^c^Hypothesis tests- Wilcoxon-Mann_Whitney test for non-normally distributed continuous data and Chi-square test for categorical data

^d^Fisher’s exact test where 1 or more cells < 5

**Fig. 4:**
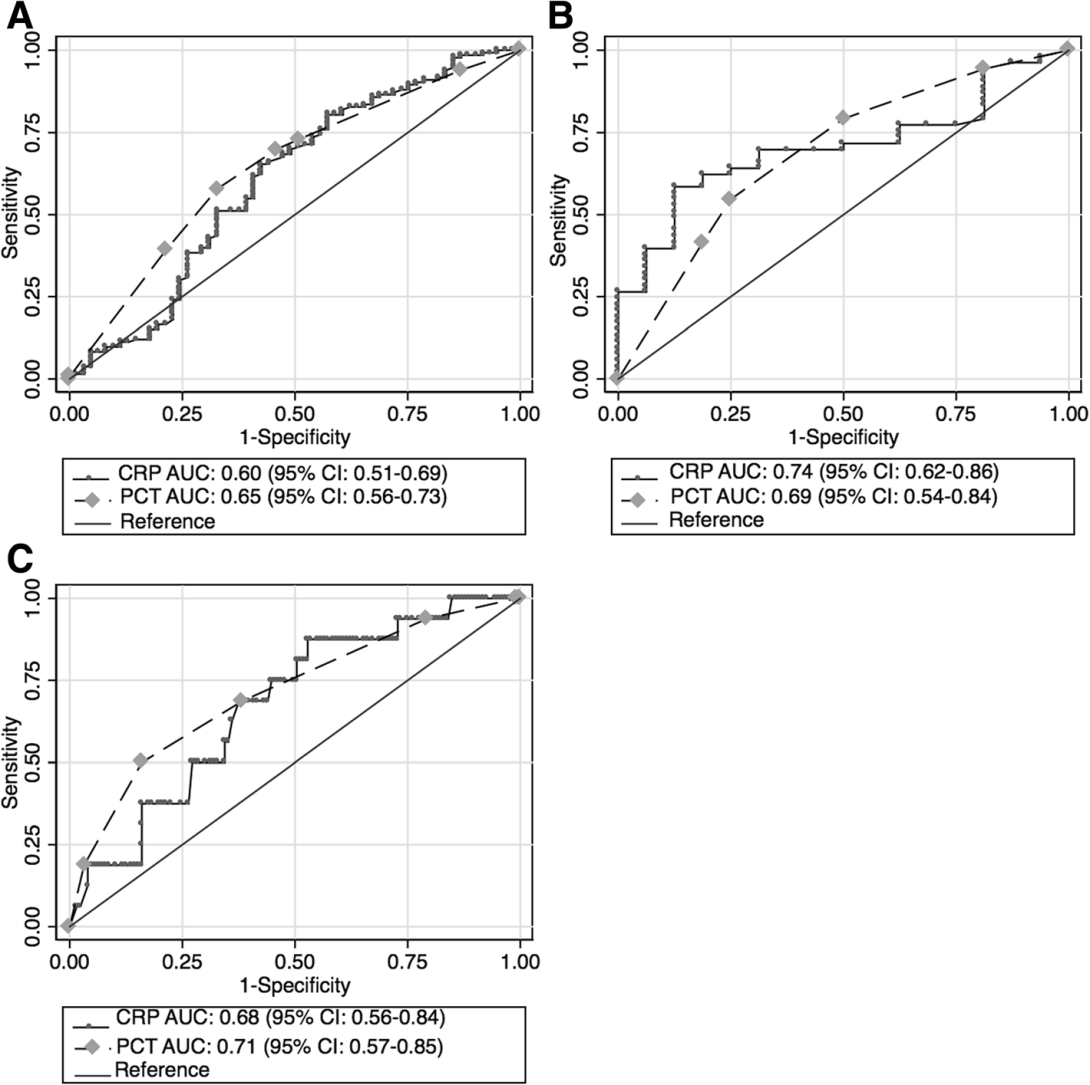
ROC curves for C-reactive protein and procalcitonin for single infection pairs. **a** Tuberculosis versus bacterial community-acquired pneumonia, (**b**) bacterial community-acquired pneumonia versus *Pneumocystis jirovecii* pneumonia, and (**c**) *Pneumocystis jirovecii* pneumonia versus tuberculosis

ROC AUCs for CRP for each single infection pair are shown in Fig. 4. Cross-validation showed minor reductions of ROC AUCs: from 0.60 to 0.58 (95% CI: 0.49–0.67) in the tuberculosis versus CAP group; from 0.74 to 0.72 (95% CI: 0.59–0.84) for CAP versus PJP; and from 0.68 to 0.64 (95% CI: 0.51–0.77) for PJP versus tuberculosis. Diagnostic accuracy estimates for two cut-offs for each infection pair and each infection versus the other two infections are presented in Table 3. CRP cut-offs with reasonable diagnostic accuracy were found for PJP versus CAP, PJP versus tuberculosis and PJP versus the other two infections.

**Table 3:** Diagnostic accuracy of C-reactive protein by infection pair in 210 participants with single target infections

Infection^a^	Cut-off (mg/L)	Sensitivity% (95% CI)	Specificity% (95% CI)	PPV% (95% CI)	NPV% (95% CI)	LR+ (95% CI)	LR- (95% CI)	Diagnostic odds ratio (95% CI)
TB vs. CAP	CRP ≥64	90.2 (83.9–94.7)	11.5 (4.7–22.2)	69.0 (61.5–75.7)	35.0 (15.4–59.2)	1.02 (0.92–1.13)	0.85 (0.36–2.03)	1.20 (0.45–3.17)
	CRP < 175	65.4 (56.7–73.4)	57.4 (44.1–70.0)	77.0 (68.1–84.4)	43.2 (32.2–54.7)	1.53 (1.12–2.11)	0.60 (0.44–0.83)	2.55 (1.37–4.72)
CAP vs. PJP	CRP ≥ 63	90.2 (79.8–96.3)	18.8 (4.0–45.6)	80.9 (69.5–89.4)	33.3 (7.5–70.1)	1.11 (0.86–1.42)	0.52 (0.15–1.87)	2.12 (0.51–8.94)
	CRP ≥ 147	63.9 (50.6–75.8)	87.5 (61.7–98.4)	95.1 (83.5–99.4)	38.9 (23.1–56.5)	5.11 (1.38–18.96)	0.41 (0.28–0.60)	12.41 (2.83–59.7)
PJP vs. TB	CRP ≥33	93.8 (69.8–99.8)	2.3 (0.5–6.5)	10.3 (5.9–16.5)	75.0 (19.4–99.4)	0.96 (0.84–1.09)	2.77 (0.31–25.08)	0.35 (0.03–3.54)
	CRP < 147	87.5 (61.7–98.4)	48.9 (40.1–57.7)	17.1 (9.7–27.0)	97.0 (89.6–99.6)	1.71 (1.33–2.19)	0.26 (0.07–0.95)	6.69 (1.46–30.60)
TB vs. CAP/PJP	CRP ≥64	90.2 (83.9–94.7)	13.0 (6.4–22.6)	64.2 (56.8–71.0)	43.5 (23.2–65.5)	1.04 (0.94–1.15)	0.75 (0.35–1.63)	1.38 (0.58–3.25)
	CRP ≥150	48.9 (40.1–57.7)	49.4 (37.8–61.0)	62.5 (52.5–71.8)	35.8 (26.8–45.7)	0.96 (0.73–1.28)	1.04 (0.78–1.37)	0.93 (0.53–1.63)
CAP vs. TB/PJP	CRP ≥63	90.2 (79.8–96.3)	10.7 (6.3–16.9)	29.3 (22.9–36.3)	72.7 (49.8–89.3)	1.01 (0.91–1.12)	0.92 (0.38–2.23)	1.10 (0.42–2.87)
	CRP ≥175	57.4 (44.1–70.0)	67.8 (59.6–75.2)	42.2 (31.4–53.5)	79.5 (71.5–86.2)	1.78 (1.30–2.45)	0.63 (0.46–0.86)	2.83 (1.54–5.21)
PJP vs. TB/CAP	CRP ≥33	93.8 (69.8–99.8)	2.6 (0.8–5.9)	7.4 (4.2–11.8)	83.3 (35.9–99.6)	0.96 (0.85–1.09)	2.42 (0.30–19.52)	0.40 (0.04–3.62)
	CRP < 147	87.5 (61.7–98.4)	53.6 (46.3–60.8)	13.5 (7.6–21.6)	98.1 (93.4–99.8)	1.89 (1.48–2.40)	0.23 (0.06–0.86)	8.09 (1.99–36.55)

Two cut-offs are listed for each infection pair and one infection versus the other two: the first with minimum 90% sensitivity and the second selected using the Liu index (see text for details)

Abbreviations: *TB* tuberculosis, *CAP* bacterial community-acquired pneumonia, *PJP Pneumocystis jirovecii* pneumonia, *PPV* positive predictive value, *NPV* negative predictive value, *LR* Likelihood ratio, *CI* confidence interval. ^a^ Cohort prevalences: TB, 63.3% (95%CI, 56.4–69.9%); CAP, 29.0% (95% CI, 23.0–35.7%); PJP, 7.6% (95% CI, 4.4–12.1%)

### Procalcitonin concentrations and diagnostic utility for each infection

Distributions of procalcitonin category in each of the three target infections are shown in Fig. 3b and Table 2. We found highest procalcitonin concentrations in the CAP group and lowest in those with PJP. There were statistically significant differences in median procalcitonin concentrations between the three infection groups, but there was marked overlap in distributions. Diagnostic performance of procalcitonin categories for discriminating between infection pairs is shown in Table 4. ROC AUCs for procalcitonin for each single infection pair are shown in Fig. 4. Best performance of procalcitonin was in discriminating between CAP and PJP.

**Table 4:** Diagnostic accuracy of procalcitonin by infection pair in 210 participants with single target infections

Infection pair^a^	Cut-off (μg/L)	Sensitivity% (95% CI)	Specificity% (95% CI)	PPV% (95% CI)	NPV% (95% CI)	LR+	LR-	Diagnostic odds ratio (95% CI)
TB vs. CAP	PCT ≥ 0.1	96.2 (91.4–98.8)	4.9 (1.0–13.7)	68.8 (61.6–75.4)	37.5 (8.5–75.5)	1.01 (0.95–1.08)	0.76 (0.19–3.10)	1.32 (0.34–5.21)
	PCT ≥ 0.25	84.2 (76.9–90.0)	18.0 (9.4–30.0)	69.1 (61.4–76.1)	34.4 (18.6–53.2)	1.03 (0.89–1.18)	0.88 (0.45–1.70)	1.17 (0.53–2.59)
	PCT ≥ 0.5	65.4 (56.7–73.4)	29.5 (18.5–42.6)	66.9 (58.1–74.9)	28.1 (17.6–40.8)	0.93 (0.76–1.14)	1.17 (0.75–1.84)	0.79 (0.41–1.52)
	PCT ≥ 2	27.1 (19.7–35.5)	50.8 (37.7–63.9)	54.5 (41.8–66.9)	24.2 (17.1–32.6)	0.55 (0.38–0.80)	1.44 (1.10–1.88)	0.38 (0.20–0.72)
	PCT > 10^b^	6.0 (2.6–11.5)	86.9 (75.8–94.2)	50.0 (24.7–5.3)	29.8 (23.2–37.1)	0.46 (0.18–1.16)	1.08 (0.97–1.20)	0.42 (0.16–1.15)
CAP vs. PJP	PCT ≥ 0.1	95.1 (86.3–99.0)	18.8 (4.0–45.6)	81.7 (70.7–89.9)	50.0 (11.8–88.2)	1.17 (0.92–1.49)	0.26 (0.06–1.180	4.46 (0.92–21.84)
	PCT ≥ 0.25	82.0 (70.0–90.6)	50.0 (24.7–75.3)	86.2 (74.6–93.9)	42.1 (20.3–66.5)	1.64 (0.99–2.71)	0.36 (0.17–0.75)	4.55 (1.44–14.43)
	PCT ≥ 0.5	70.5 (57.4–81.5)	56.2 (29.9–80.2)	86.0 (73.3–94.2)	33.3 (16.5–54.0)	1.61 (0.90–2.87)	0.52 (0.29–0.94)	3.07 (1.02–9.26)
	PCT ≥ 2	49.2 (36.1–62.3)	81.2 (54.4–96.0)	90.9 (75.7–98.1)	29.5 (16.8–45.2)	2.62 (0.92–7.51)	0.63 (0.44–0.88)	4.19 (1.15–15.03)
PJP vs. TB	PCT ≥ 0.1	81.2 (54.4–96.0)	3.8 (1.2–8.6)	9.2 (5.0–15.3)	62.5 (24.5–91.5)	0.84 (0.67–1.07)	4.99 (1.31–18.94)	0.17 (0.04–0.71)
	PCT ≥ 0.25	50.0 (24.7–75.3)	15.8 (10.0–23.1)	6.7 (2.9–12.7)	72.4 (52.8–87.3)	0.59 (0.36–0.97)	3.17 (1.69–5.93)	0.19 (0.07–0.54)
	PCT ≥ 0.5	43.8 (19.8–70.1)	34.6 (26.6–43.3)	7.4 (3.0–14.7)	83.6 (71.2–92.2)	0.67 (0.38–1.18)	1.63 (1.00–2.66)	0.41 (0.15–1.14)
	PCT ≥ 2	18.8 (4.0–45.6)	72.9 (64.5–80.3)	7.7 (1.6–20.9)	88.2 (80.6–93.6)	0.69 (0.24–1.99)	1.11 (0.86–1.44)	0.62 (0.18–2.17)

Selected categories are based on assay guidelines developed for antibiotic use guidance in lower respiratory tract infections and sepsis (see text for details)

Abbreviations: *TB* tuberculosis, *CAP* bacterial community-acquired pneumonia, *PJP Pneumocystis jirovecii* pneumonia, *PPV* positive predictive value, *NPV* negative predictive value, *LR* Likelihood ratio, *CI* confidence interval. ^a^Cohort prevalences: TB, 63,3% (95%CI, 56.4–69.9%); CAP, 29.0% (95% CI, 23.0–35.7%); PJP, 7.6% (95% CI, 4.4–12.1%)

^b^Analaysis of PCT category of > 10 was only performed in TB vs. CAP infection pairs as no PJP participants had PCT exceeding 10 μg/L

### Participants with mixed infection

Analysis of baseline characteristics and biomarker concentrations for those with mixed infection compared with those with single target infections are summarised in an additional table (see Additional file 2). Analysis of both biomarkers showed wide distributions, overlapping with those of the three mono-infections. Elevated CRP was found in all 38 participants with mixed infections. Both CRP and procalcitonin medians were statistically higher in the mixed infection group compared with the PJP group. Three participants had procalcitonin concentrations < 0.1 μg/L, all of whom had CAP dual infection (two with tuberculosis and the other with PJP). Procalcitonin ≥0.25 μg/L captured 29/37 (78.4) with CAP dual infection, ≥ 0.5 μg/L captured 23/37 (62.2%), and 14 (37.8%) exceeded the ≥2 μg/L cut-off, 3 of whom had concentrations above 10 μg/L.

## Discussion

We evaluated the diagnostic accuracy of CRP and procalcitonin for differentiating between the three major infections affecting HIV-infected adult inpatients. Our study is one of only a very few to assess the diagnostic accuracy of CRP and procalcitonin for the three commonest infections in HIV-infected inpatients. We found statistically significant differences in median CRP and procalcitonin concentrations between the three infection groups, but there was marked overlap in distributions. Participants with PJP had lower CRP and procalcitonin concentrations. Procalcitonin and CRP had ROC AUCs of around 0.7 for discriminating PJP from CAP and tuberculosis in pairwise comparisons, indicating moderate discrimination, but both biomarkers performed less well in discriminating CAP from tuberculosis. A CRP cut-off of 147 mg/L had high specificity for discriminating PJP from CAP, and high sensitivity for discriminating PJP from tuberculosis. We found cut-offs with sensitivities of 90% or more for CRP for all three target infection pairs, and for procalcitonin for two target infection pairs (tuberculosis versus PJP and CAP versus PJP), but specificities were much lower than the 70% recommended by WHO for tuberculosis screening tests [20]. Our findings suggest that CRP and/or procalcitonin should be explored in the development of clinical prediction rules in seriously ill HIV-infected patients or in a panel of biomarkers rather than be used as stand alone diagnostic tests.

Previous studies have demonstrated the diagnostic value of CRP in active tuberculosis case detection in otherwise healthy HIV-infected persons, however higher false positive rates were found in passive case detection [8]. Elevated CRP is known to occur in all three of our target infections in individuals with HIV, and our finding that CRP concentrations were highest in CAP, follwed by tuberculosis, and lowest in PJP, is consistent with previous studies [12, 13, 22]. We found that diagnostic performance of CRP in our study population was limited by widely overlapping distributions between the three target infections, resulting in reduced utility for inpatient populations where these are the three commonest competing aetiologies. Similar low specificity for discriminating between all three infections was shown in a British case notes review study of HIV-infected adults admitted with respiratory infections [13]. Conversely, our findings differed from a previous South African study that reported good discrimintaion of CRP (ROC AUC of 0.87) when comparing participants with pneumococcal community-acquired pneumonia and pulmonary tuberculosis [22]. We suspect that the disparity may be attributable to differences in participant selection or to the small sample size in the other South African study. Another study found specificity (83%) and sensitivity 69%, for combined CRP and IL-8 for discriminating bacerial pneumonia from PJP or mycobateriosis, in a cohort that included hospital-acquired infections and non-tuberculous mycobacterial infections [12].

Guidelines for procalcitonin suggest that bacterial infection is present at a concentration of ≥0.25 μg/L [9]. Although this cut-off captured 82% of participants with CAP in our study, it also identified 84% of those with tuberculosis and 50% of those with PJP; therefore had limited value in distinguishing CAP from the other two infections. Procalcitonin distribution across the three infections in our cohort, with concentrations highest in CAP and lowest in PJP, were comparable to other studies in HIV-infected individuals [22, 23]. However, our findings did not mirror those of two other studies of inpatients with mixed HIV status, both of which found little overlap in procalcitonin concentrations between any of the three target infections [22, 23]. In Schleicher’s study, elevated procalcitonin (> 0.1 μg/L) was found in all participants with bacterial pneumonia and only 59% of the tuberculosis group, compared with 95% and 96% respectively in our study. Schleicher et al. and Lawn et al. noted a possible link between raised procalcitonin and lower CD4 count [20, 22]; in our participants with tuberculosis as a single infection we also found a moderate negative association between CD4 count and procalcitonin (Spearman’s rho − 0.33, *p*-value = 0.0001). Differing CD4 count medians (107 in Schleicher’s cohort compared with 77 × 10^9^ cells/L in ours) tuberculosis versus PJP and CAP versus PJP may account for higher procalcitonin concentrations in our tuberculosis group.

Mixed infection is a well-recognised limitation in diagnostic accuracy studies. Our study prevalence of 15.3% mixed infection compared to Nyamande’s reported 21% [23]. In our study, elevated CRP was found in all 38 participants with mixed infection and elevated procalcitonin in 92%. Our participants with mixed infection had higher median concentrations of both CRP and procalcitonin than the participants with tuberculosis and PJP as single infections, but statistical significance was only found when comparing mixed infection to PJP as a single infection. Due to the difficulty in determining the extent of contribution of each infection to biomarker concentrations, the mixed infection group was excluded from diagnostic accuracy analyses.

Our study has a number of limitations. First, reference standards for CAP and PJP did not include microbiological confirmation. Although blood and sputum cultures were carried out to detect bacterial infections, almost all were negative due to prior antibiotic use. However, even if cultures had been taken prior to antibiotics the sensitivity of sputum and blood cultures for CAP is low, and clinical case definitions of CAP are universally used in clinical research. Bronchoalveolar lavage, which is the optimal specimen for diagnosing PJP, was not available at either of our study hospitals. However, we adapted the CDC case definition for PJP, which has a sensitivity and specificity of 85% for diagnosis of PJP when compared with bronchoscopy [24]. Furthermore, we found β-D-glucan exceeding 300 pg/L in 80% of our participants fulfilling our PJP case definition, compared with 8% for tuberculosis and 2% for CAP. β-D-glucan had a sensitivity of 92% and a specificity of 78% for the diagnosis of PJP in HIV-infected patients in a systematic review [25], and 91% sensitivity and 92% specificity for a β-D-glucan cut-off > 300 pg/L in one study [26]. Second, our sample size of participants with PJP was small. Third, CRP and procalcitonin concentrations may have been reduced in participants with bacterial infections by antibiotic treatment prior to providing a blood specimen at study enrolment, which would reduce the ability of the biomarkers to discriminate CAP from TB and PJP. However, this is unlikely to have had a major effect as almost all of our participants received antibiotics within 24 h of study enrolment. Study strengths include the use of a robust culture-based reference standard for tuberculosis and that our study participants were recruited from two urban district hospitals that represent a population typical of high HIV and tuberculosis burden settings.

## Conclusions

CRP and procalcitonin were both found to have limited value in distinguishing between the three common infections due to widely overlapping distributions, particularly between tuberculosis and CAP. Future studies should include a larger sample of participants with PJP definitively diagnosed, as both biomarkers had best diagnostic accuracy for discriminating between PJP and the other two infections in our study. CRP and procalcitonin may have greater diagnostic utility as part of a panel of biomarkers or in clinical prediction rules.

## Additional files

Additional file 1: Power calculations. **Table S1.** Power calculation for sensitivity estimates for each target infection. **Table S2.** Power calculation to detect C-reactive protein and procalcitonin concentration differences between target disease pairs (DOCX 19 kb)

Additional file 2: Baseline characteristics including participants with mixed infection. (PDF 105 kb)

## Abbreviations

AFB: Acid fast bacilli
AUC: Area under the curve
BDL: Below the detectable limit
CAP: Bacterial community-acquired pneumonia
CI: Confidence interval
CRP: C-reactive protein
HIV: Human immunodeficiency virus
LRTI: Lower respiratory tract infection
PJP: *Pneumocystis jirovecii* pneumonia
ROC: Receiver operating characteristic
STARD: Standards for reporting diagnostic accuracy studies
WHO: World Health Organization

## References

[1] Ford N, Shubber Z, Meintjes G, Grinsztejn B, Eholie S, Mills EJ (2015). Causes of hospital admission among people living with HIV world-wide: a systematic review and meta-analysis. Lancet HIV.

[2] Feldman C, Brink AJ, Richards GA, Maartens G, Bateman ED (2007). Management of community-acquired pneumonia in adults. Working group of the South African Thoracic Society. SAMJ.

[3] Chegou NN, Hoek KGP, Kriel M, Warren RM, Victor TC, Walzl G (2011). Tuberculosis assays: past, present and future. Expert Rev Anti-Infect Ther.

[4] Trebucq A, Enarson DA, Chiang CY, Van Deun A, Harries AD, Boillot F (2011). Xpert® MTB/RIF for national tuberculosis programmes in low-income countries: when, where and how?. Int J Tuberc Lung Dis..

[5] Garcia-Vazquez E, Marcos MA, Mensa J, de Roux A, Puig J, Font C (2004). Assessment of the usefulness of sputum culture for diagnosis of community-asquired pneumonia using the PORT predictive scoring system. Arch Intern Med.

[6] World Health Organization. Consolidated guidelines on the use of antiretroviral drugs for treating and preventing HIV infection. Recommendations for a public health approach. Second edition. http://www.who.int/hiv/pub/arv/arv-2016/en/ Accessed 4 Oct 2017.

[7] Griesel R, Stewart A, van der Plas H, Sikhondze W, Rangaka MX, Nicol MP (2018). Optimizing tuberculosis diagnosis in human immunodeficiency virus-infected inpatients meeting the criteria of seriously ill in the World Health Organisation. Clin Infect Dis.

[8] Yoon C, Chaisson LH, Patel SM, Allen IE, Drain PK, Wilson D (2017). Diagnostic accuracy of C-reactive protein for active pulmonary tuberculosis: a meta-analysis. Int J Tuberc Dis.

[9] Scheutz P, Chiappa V, Briel M, Greenwald L (2011). Procalcitonin algorithms for antibiotic therapy decisions. A systematic review of randomized controlled trials and recommendations for clinical algorithms. Arch Intern Med.

[10] Yoon C, Simitala FC, Atuhumoza E, Katende J, Mwebe S, Asege L, et al. Point-of-care C-reactive protein-based tuberculosis screening for people living with HIV: a diagnostic accuracy study. The Lancet online 2017. 10.1016/S1473-3099(17)30488-7. Accessed 8 Dec 2017. 10.1016/S1473-3099(17)30488-7 PMC5705273 28847636

[11] Corti C, Fally M, Fabricius-Bjerre A, Mortensen K, Jensen BN, Andreassen HF (2016). Point-of-care procalcitonin test to reduce antibiotic exposure in patients hospitalized with acute exacerbation of COPD. Int J Chron Obstruct Pulmon Dis.

[12] Benito N, Moreno A, Filella X, Miro MJ, Gonzalez J, Pumarola P (2004). Inflammatory responses in blood samples of human immunodeficiency virus-infected patients with pulmonary infections. Clin Diagn Lab Immunol.

[13] Sage EK, Noursadeghi M, Evans HE, Noursadeghi M, Parker SJ, Copas AJ (2010). Prognostic value of C-reactive protein in HIV-infected patients with *Pneumocystis jirovecii* pneumonia. Int J STD AIDS.

[14] Scott JAG, Hall AJ, Muyodi C, Lowe B, Ross M, Chohan B (2000). Aetiology, outcome, and risk factors for mortality among adults with acute pneumonia in Kenya. Lancet.

[15] Centers for Disease Control. Revision of the CDC surveillance case definition for Acquired Immunodeficiency Syndrome. Morbidity and mortality weekly report. 1987; 36(suppl no1S): Appendix III p. 13S. https://www.cdc.gov/mmwr/pdf/other/mmsu3601.pdf Accessed 10 Jan 2017.

[16] Agresti A, Coull BA (1998). Approximate is better than “exact” for interval estimation of binomial proportions. Am Stat.

[17] Lehmann EL. Nonparametrics: statistical methods based on ranks, revised. Upper Saddle River: Prentice Hall, Inc; 1998. p. 76–81.

[18] LaFleur B, Lee W, Merchant N (2011). Statistical methods for assays with limits of detection: serum bile acid as a differentiator between patients with normal colons, adenomas, and colorectal cancer. J Carcinog.

[19] Liu X (2012). Classification accuracy and cut point selection. Stat Med.

[20] World Health Organization. High-priority target product profiles for new tuberculosis diagnostics: report of a concensus meeting. http://www.who.int/tb/publications/tpp_report/en/ Accessed 16 Jun 2017.

[21] Bossuyt PM, Reitsma JB, Bruns DE, Gatsonis CA, Glasziou PP, Irwig L, et al. For the STARD Group. STARD 2015: an updated list of essential items for reporting diagnostic accuracy studies. BMJ. 2015;351:h5527. 10.1136/bmj.h5527 PMC4623764 26511519

[22] Schleicher GK, Herbert V, Brink A, Martin S, Maraj R, Galpin JS (2005). Procalcitonin and C-reactive protein levels in HIV-positive subjects with tuberculosis and pneumonia. Eur Respir J.

[23] Nyamande K, Lalloo UG (2006). Serum procalcitonin distinguishes CAP due to bacteria, *Mycobacterium tuberculosis* and PJP. Int J Tuberc Lung Dis.

[24] Miller RF, Millar AB, Weller IVD, Semple SJG (1989). Empirical treatment without bronchoscopy for *Pneumocystis carinii* pneumonia in the acquired immunodeficiency syndrome. Thorax.

[25] Li WJ, Guo YL, Liu TJ, Wang K, Kong JL (2015). Diagnosis of *Pneumocystis* pneumonia using serum (1-3)-β-D-glucan: a bivariate meta-analysis and systematic review. J Thorac Dis.

[26] Salerno D, Mushatt D, Myers L, Zhuang Y, de la Rua N, Calderon EJ (2014). Serum and bal beta-D-glucan for the diagnosis of *Pneumocystis* pneumonia in HIV postive patients. Resp Med.

