# Supplementary material for: C-reactive protein and procalcitonin to discriminate between tuberculosis, Pneumocystis jirovecii pneumonia, and bacterial pneumonia in HIV-infected inpatients meeting WHO criteria for seriously ill: a prospective cohort study
Source: BMC Infect Dis. 2018 Aug 14;18:399. doi: 10.1186/s12879-018-3303-6 (PMC6092834; doi:10.1186/s12879-018-3303-6)
Supplement: Supplementary file 1 — Power calculations. Table S1. Power calculation for sensitivity estimates for each target infection. Table S2. Power calculation to detect C-reactive protein and procalcitonin concentration differences between target disease pairs (DOCX 19 kb) [file 12879_2018_3303_MOESM1_ESM.docx]

**Additional File 1: Power calculations**

**Table S1:** Power calculation for sensitivity estimates for each target infection

| **% CI** | **95% CI** | | | **99% CI** | | |
| --- | --- | --- | --- | --- | --- | --- |
| **Sensitivity** | **70%** | **80%** | **90%** | **70%** | **80%** | **90%** |
| **TB** |  |  |  |  |  |  |
| n=133 | 61.66-77.07 | 72.07-85.66 | 84-94.2 | 69.41-87.17 | 69.41-87.17 | 81.56-95.06 |
| n=113 | 60.91-77.6 | 71.31-86.04 | 83.41-94.48 | 68.38-87.63 | 68.38-87.63 | 80.7-95.36 |
| **CAP** |  |  |  |  |  |  |
| n=61 | 58.11-80.44 | 68.69-88.37 | 80.16-95.41 | 54.06-82.91 | 64.55-90.15 | 76.1-96.35 |
| n=52 | 55.73-80.09 | 68.10-89.2 | 79.4-95.82 | 51.38-82.73 | 63.58-91 | 74.9-96.3 |
| **PJP** |  |  |  |  |  |  |
| n=16 | 44.4-85.84 | 57- 93.4 | 64-95.5 | 37.56-88.94 | 49.06- 95.12 | 55.5-97.52 |
| n=14 | 45.35-88.28 | 52.41-92.42 | 68.53-98.73 | 38.01-91.07 | 44.37-94.4 | 59-99.16 |
| Abbreviations: CI: confidence intervals, TB: tuberculosis, CAP: bacterial community-acquired pneumonia, PJP: *Pneumocystis jirovecii* pneumonia. | | | | | | |

**Table S2**: Power calculation to detect C-reactive protein and procalcitonin concentration differences between target disease pairs

| **Test** | **Reference mean** | **Absolute differences across all ranges** | | **Approximate**  **effect size (%**  **difference)** | **Approximate power (%) with different error probabilities & sample sizes** | | | |
| --- | --- | --- | --- | --- | --- | --- | --- | --- |
|  |  |  |  |  | **100% n (TB:133**  **CAP:61, PJP:16)** | | **85% n (TB:113, CAP:52, PJP:14)** | |
| **PCT** | **ng/ml (95% CI)** | |  |  | **0.05** | **0.10** | **0.05** | **0.10** |
| **TB vs.**  **PJP** | 4.164  (1.749-6.579)  1.138  (0.543-1.734) | 6  2.1  2  0.02 | | 92  50  48  0.009 | 100  88  85  - | 100  94  92  - | 100  83  79  - | 100  91  88  - |
|  |  |  | |  |  |  |  |  |
| **CAP vs.**  **PJP** | 19.479  (8.02-30.94)  1.138  (0.543-1.734) | 19  18  12  10  6 | | 97  94  62  51  78 | 100  99  86  74  39 | 99  99  81  84  54 | 99  99  81  67  35 | 100  100  89  80  50 |
|  |  |  | |  |  |  |  |  |
| **CAP vs.**  **TB** | 19.479  (8.02-30.94)  4.164  (1.749-6.579) | 29  15  12  11  1 | | 94  79  62  56  0.05 | 100  96  87  80  - | 100  98  93  89  15 | 100  93  81  75  - | 100  97  90  85  14 |
| **CRP** | **mg/L (SD)** |  | |  | **100%**  **0.05** | **100%**  **0.10** | **85%**  **0.05** | **85%**  **0.10** |
| **TB**  **vs.**  **PJP** | 98.7 (48.3)  47.0 (49.0) | 52  36  30 | | 53  36  30 | 99  85  72 | 100  92  84 | 97  80  67 | 99  89  80 |
| **CAP**  **Vs.**  **PJP** | 217.0 (16.0)  47.0 (49.0) | 78  35  30 | | 36  16  14 | 100  85  75 | 100  93  86 | 100  80  69 | 100  90  82 |
| **CAP**  **Vs.**  **TB** | 217.0 (16.0)  98.7 (48.3) | 55  35  30 | | 25  16  14 | 100  85  75 | 100  93  86 | 99  80  70 | 100  90  82 |
| Abbreviations: CI: confidence intervals, TB: tuberculosis, CAP: bacterial community-acquired pneumonia, PJP: *Pneumocystis jirovecii* pneumonia. | | | | | | | | |
