# Supplementary material for: C-reactive protein and procalcitonin to discriminate between tuberculosis, Pneumocystis jirovecii pneumonia, and bacterial pneumonia in HIV-infected inpatients meeting WHO criteria for seriously ill: a prospective cohort study
Source: BMC Infect Dis. 2018 Aug 14;18:399. doi: 10.1186/s12879-018-3303-6 (PMC6092834; doi:10.1186/s12879-018-3303-6)
Supplement: Supplementary file 2 — Baseline characteristics including participants with mixed infection. (PDF 105 kb) [file 12879_2018_3303_MOESM2_ESM.pdf]

Additional File 2: baseline characteristics including participants with mixed infection.

Table 1: Baseline characteristics of 248 participants with a single target infection or mixed infection by infection status

Diagnosis (%)	Total N=248	TB n=133 (54)	CAP n=61 (25)	PJP n=16 (6)	Mixed infection n= 38 (15)	P-value for pairwise comparison*
**Median age in yrs. (IQR)**	34.7 (29.1-41.2)	34.7 (29.1-40.1)	35.1 (29.4-40.0)	36.9 (28.8-41.2)	34.5 (29.9-43.8)	TB vs. MI=0.66, CAP vs. MI=0.74, PJP vs. MI=0.91
**Sex: female n(%)**	168 (68)	84 (63)	44(72)	11(69)	29(76)	TB vs. MI=0.13, CAP vs. MI=0.65 PJP vs. MI= 0.57
**Cotrimoxazole prophylaxis**	73 (29)	38(29)	18(30)	4(25)	13(34)	TB vs. MI=0.50, CAP vs. MI=0.63 PJP vs. MI= 0.75#
**Antiretroviral therapy n (%)**	93 (38)	43(32)	29(48)	4(25)	17(45)	TB vs. MI=0.16, CAP vs. MI=0.79 PJP vs. MI=0.23#
**Median $CD4^+$ count, cells/µL (IQR)**	94 (36-210)	77 (35-162)	199.5 (78.5-287)	35 (11.5-80.5)	79.5 (23-202)	TB vs. MI=0.9, CAP vs. MI=0.004 PJP vs. MI=0.17
**Median WCC $x10^9$/L (IQR)**	8.9 (5.8-12.9)	7.3 (5.2-10.2)	12.3 (8.4-20.0)	8.2 (6.2-10.7)	10.3 (7.2-14.8)	TB vs. MI=0.005, CAP vs. MI=0.10 PJP vs. MI=0.16
**Median Hb g/dl (IQR)**	9.4 (7.7-10.9)	8.6 (7.4-10.1)	10.4 (8.8-12)	11.25 (9.7-12.2)	9.4 (7.8-11.6)	TB vs. MI=0.05, CAP vs. MI=0.14 PJP vs. MI=0.05
**Median CRP mg/L (IQR)**	149.5 (92-223.5)	141 (97-203)	193 (108-264)	106.5 (79.5-131.5)	184.5 (82-223)	TB vs. MI: 0.52, CAP vs. MI: 0.25 PJP vs. MI: 0.03
**Median PCT µg/L (IQR)**	0.8 (0.3-3.2)	0.7 (0.4-2.1)	2.0 (0.4-5.2)	0.2 (0.1-1.3)	0.7 (0.3-4.2)	TB vs. MI: 0.96, CAP vs. MI: 0.26 PJP vs. MI: 0.09
**PCT≥0.25 µg/L n(%)**	200 (80.7)	112 (84.2)	50 (82.0)	8 (50.0)	30 (79.0)	PTB vs. MI: 0.45, CAP vs. MI: 0.71 PJP vs. MI: 0.03
**WHO danger signs[1]:**						
**Pulse rate> 120beats/min[1]**	199 (80)	106 (80)	51 (84)	9 (56)	33(87)	TB vs. MI=0.32, CAP vs. MI=0.66 PJP vs. MI= 0.01
**Respiratory rate>30/min[1]**	162(65)	83 (62)	38 (62)	16 (100)	25(66)	TB vs. MI=0.70, CAP vs. MI=0.73 PJP vs. MI=0.006#
**Temperature >39°C[1]**	38 (15)	20 (15)	10 (16)	1 (6)	7 (18)	TB vs. MI=0.61, CAP vs. MI=0.80 PJP vs. MI= 0.25
**Unable to walk unaided[1]**	140 (56)	88 (67)	23 (38)	8 (50)	21(55)	TB vs. MI=0.20, CAP vs. MI=0.10 PJP vs. MI=0.72

Abbreviations: TB, tuberculosis; CAP, bacterial community-acquired pneumonia; PJP, *Pneumocystis jirovecii* pneumonia; MI: mixed infection; Hb: haemoglobin; WCC: white cell count; CRP, C-reactive protein; PCT, procalcitonin

*Hypothesis tests- Wilcoxon-Mann-Whitney test for continuous data; Chi-square test for categorical data. #Fisher's exact test where 1 or more cells <5

[1] Danger signs based on WHO algorithm for diagnosis of TB in seriously ill patients.
